# Zoning for management in wetland nature reserves: a case study using Wuliangsuhai Nature Reserve, China

**DOI:** 10.1186/2193-1801-1-23

**Published:** 2012-10-02

**Authors:** Qing Zeng, Yamian Zhang, Yifei Jia, Shengwu Jiao, Duoduo Feng, Peter Bridgewater, Guangchun Lei

**Affiliations:** 1 Beijing Forestry University, P.O. Box 159, Beijing, 100083 China; 2 Beijing Forestry University, P.O. Box 963, Beijing, 100083 China

**Keywords:** Wetland, Management, Nature reserve, Zonation, Wuliangsuhai, Water birds, Ecological character, Ramsar Convention

## Abstract

**Background:**

Zoning is a fundamental tool for the effective management of nature reserves. A three-zone model (core zone, buffer zone, and experimental zone) has been applied to nature reserves in China since 1980s; however, this model appears not fit for all types of nature reserves, especially wetlands.

**Case description:**

Wuliangsuhai is such a typical wetland reserve, which can represent most of the other wetland reserves in China, for both its human utilization, and for its function as the bird habitat. The “Component-Process-Service” (CPS) framework of the Convention on Wetlands allows a determination of the “ecological character” of the wetland and also allows identification of potential threats, providing thus a perspective for management opportunities and challenges.

**Discussion and evaluation:**

Applying the CPS framework to Wuliangsuhai wetland nature reserve, we have had a better understanding of the ecosystem services and its relationship with the ecological process and components of the wetland. A comparison of effectiveness in maintaining ecosystem services by the two zoning models (the existing three-zone model, and the new zoning model) was made.

**Conclusions:**

The study suggested introducing an additional risk-control zone to be more effective in managing and alleviating threats to the ecological character than the standard 3-zone system. Furthermore, a “dynamic” zoning that takes into account the annual variation in habitat and avifauna distribution, as an elaboration of the Four-zone structure, may achieve the desired conservation objectives in an even more effective manner. The proposed zonation structure has the added benefit of promoting harmonization between nature conservation and local sustainable development.

## Background

As a means of comparing climatic and altitudinal variation, as well as providing insights into temporal changes (such as succession), the study of ecosystem zonation has a long history. As a natural area management tool, however, zonation has a shorter history with its origins dating back to the 1930s. At that time, when the need for management of wild landscapes as protected areas was beginning to be understood, dividing their space into areas of different management activities and intensities became common practice. Shelford (1933) among others, proposed establishment of buffer zones to keep innermost cores of protected areas from human interference. It is since the 1960’s that zoning as a management tool has proceeded apace in the management of public land, fisheries, and especially marine, terrestrial and freshwater protected areas (Day 2002, Davos *et al*. 2007, Salomon *et al*. 1996). Well-designed and scientifically-based zonation can be indeed a useful and important way to allocate management effort and attention, define appropriate levels of enforcement, reconcile different users’ conflicts and establish appropriate monitoring protocols.

Perhaps the most widespread global standard for protected area zonation is that of UNESCO Biosphere Reserves (UNESCO 1970). Part of the acceptance process to be included in the World Network of Biosphere Reserves was the need to comply with a tripartite zonation: core area, buffer zone, and transition area. China has adopted a version of this 3-zone plan as an essential principle for design and management of not only its Biosphere Reserves but all its nature reserves. Current data show China has 2,541 nature reserves, covering almost 150 million ha, and representing 15.3% of land surface, of which wetland reserves (excluding coastal wetlands) accounted for 19% (MEPPRC 2010). Yet in the 30 years during which wetland reserves have been established in China, and despite advances in technology and methods of mapping and observation, the model and theory behind reserve zonation has remained unchanged - and unchallenged.

Unlike other ecosystems e.g. forests, dry grasslands, savannah forests, management challenges in wetland nature reserves arise from the use of the reserve by transitory species (e.g. migratory water birds), which suggested that a three-zone management scheme is not the most effective or efficient zonation framework. Among the reasons for lack of effectiveness are the highly dynamic characteristics of wetlands - including seasonal climatic and hydrological variation, against which inflexible conservation objectives and strategies provide problems, rather than solutions.

In this study, we applied the ecological character paradigm (Ramsar 2005) of the Convention on Wetlands (Ramsar 1971) – hereafter Ramsar Convention - to understand the spatial and temporal nature of the wetland in the Wuliangsuhai Nature Reserve (hereafter WNR), China. We were not only mindful of the static ecological components such as vegetation distribution, habitat types, but also of ecological processes (birds migratory pattern, hydrology, etc.) and the delivery of welfare for local people (wetland ecosystem services), to get a refined zonation model as a compromise of conservation and development.

## Case description

### Study area

**Figure 1:**
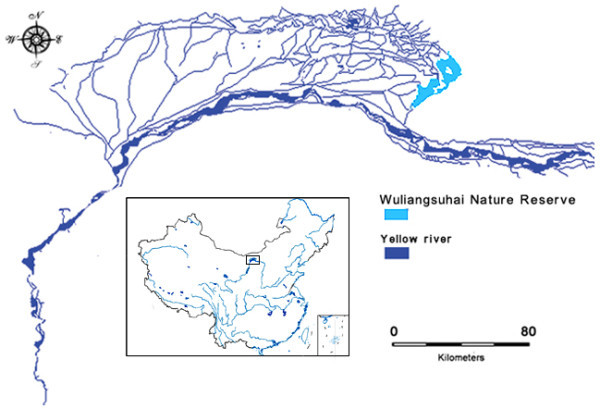
**Location of Wuliangsuhai Nature Reserve.**

The study area (WNR) is located at 40°47’-41°03’N, 108°43’-108°57’E, on the eastern Ho-t'ao Plain in western Inner Mongolia (Figure 1). The key part is a large shallow lake covering an area of 293 km^2^, the largest water body associated with the Yellow River in Inner Mongolia. In the past 150 years, partly as a result of sediment loads in the Yellow river raising the river bed above surrounding lands, the Alxa desert has moved eastwards. This eastward movement caused the eventual isolation of Wuliangsuhai Lake in an area otherwise dominated by semi-arid grassland. With a maximum depth of 2.5 m, the average water level was 1.12 m, but the area with a water level less than 0.7 m accounted for 85% of the total lake surface, which allows extensive reed (*Phragmites australis*) swamp develop (Wang and Dou 1998).

Before the establishment of the nature reserve, the lake had been heavily used as the fish farm by local communities. Their livelihoods had been depending on the revenue from fishery, and harvest of reeds that sell to nearby paper mill. Since the area been declared as the nature reserve, such activities should be regulated by law, however, due to lack of compensation mechanism, all these illegal activities continue, in addition, tourism has been developed in recent years. Thus, the conflict between conservation and maintaining livelihoods has been growing.

The WNR lies in the conjunction of the Central Asian Flyway and the East Asian-Australian Flyway and represents one of the most important breeding and stopover sites on these flyways (Boere & Stroud 2006). WNR has had 240 species of birds from 46 families and 17 orders recorded from its waters. Among those 240 species are 5 first class protected species and 29 second class protected species, according to Chinese Wildlife Conservation Legislation. Particularly important species include *Ciconia nigra*, *Haliaeetus leucoryphys*, *Haliueetus albiilla*, *Otis tarda, Larus relictus*, and, the most emblematic, *Cygnus olor* (Mute Swan)*.*

**Figure 2:**
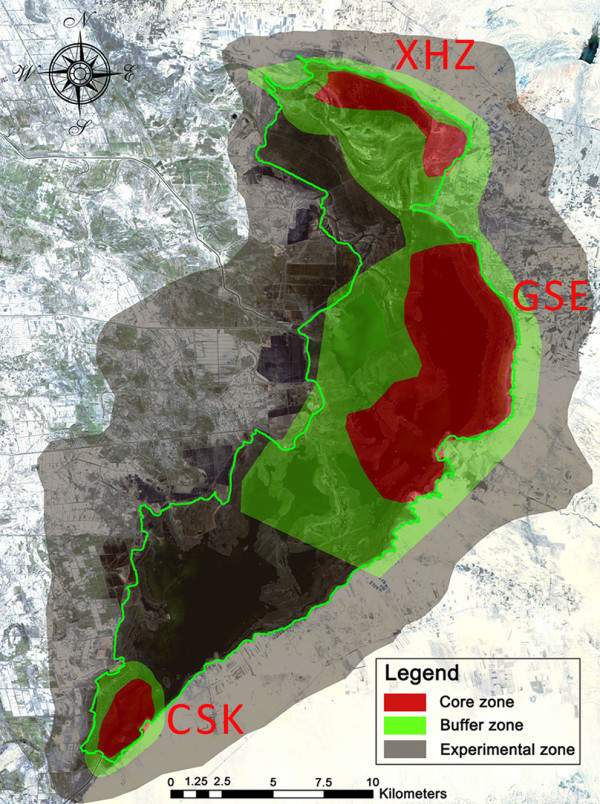
**Current zonation of Wuliangsuhai Nature Reserve.** Red area equals the core zone, light-green area equals the buffer zone, and yellow area equals the experimental zone. The boundary of Wuliangsuhai wetland is depicted by a solid green line.

WNR was set up in 1993 as a city-level reserve with water bird fauna and wetland ecosystems explicitly as its key conservation targets. In 1998 it became a provincial level reserve with an area of 600 km^2^, retaining the original targets and the traditional three-zone structure for management. The core zone was established in three parts (Figure 2): a site in the south of the wetland, Chu ShuiKou (CSK); a middle site, GeSuer (GSR) and a site to the north, Xiao HaiZi (XHZ), with areas of 18 km^2^, 60 km^2^ and 15 km^2^ respectively. There were 0.5-1.0 km buffers are around the core zones, taking up 120 km^2^, almost 20% of the whole area. These “core-buffer” systems were encased in a large experimental zone taking up 387 km^2^ or 64.5% of the area.

### The ecological character paradigm

The Ramsar Convention defines ecological character (prescribed by the Convention text in Article 3) and adopted in revised form by Decision IX.1A (Ramsar 2005) as: 

*"“… the combination of the ecological components, processes and ecosystem services that characterize the wetland.”"*

Ecological components are further defined as the *physical, chemical and biological(communities, habitats, species, genes)elements of a wetlandecosystem*. Ecological Processes are the *dynamic interactions within andbetween the biotic components,as well as betweenthem and with theabiotic components*. Ecosystem Services - usually grouped as provisioning, regulating, cultural and supporting (MA [2003]) - are provided by the wetland ecosystem *through interactions between thecomponents and dynamic ecologicalprocesses.*

**Figure 3:**
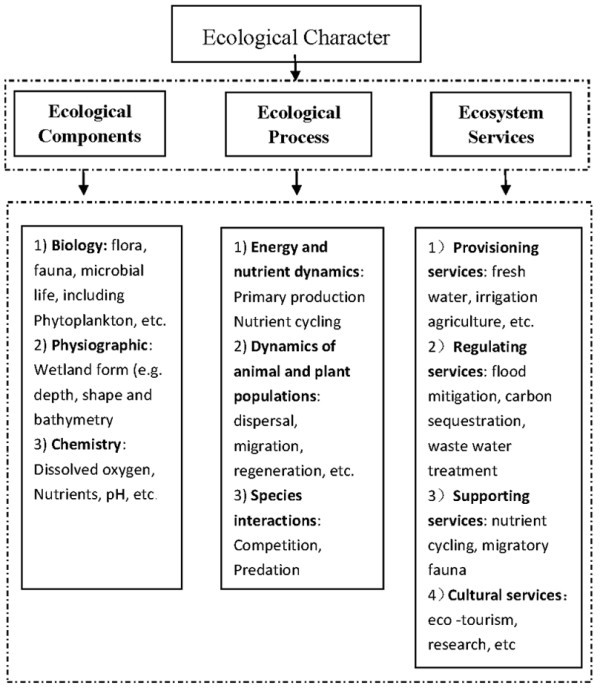
**Ecological Character as currently defined (Resolution IX.1A) by the Ramsar Convention.**

This work has given rise to the Component-Process-Services (CPS) conceptual framework, adopted by the Ramsar Convention (Ramsar 2005,2008) to aid description of the ecological character of a wetland (Figure 3). This CPS framework has been used as the primary organising structure for the investigation of biodiversity baseline. Several different methods and techniques were used in the analysis of components.

Vegetation and habitat distribution of key structural types was derived from an image-based survey using Alos Imagery data (2010-10-14) with 2.5 m resolution. Analysis of the image data using ArcGIS 9.3 and ENVI 4.7 produced area calculations.

For Bird species, we analysed, through a three-year (2009–2011) investigation from spring to winter - water bird population distribution and dynamics in the WNR. Plot count and the line transect methods were used. Each survey consists of 2 plots and 5 transects sets and were conducted by a 3-member team. The plots and transects covered the three different habitats (open water, reed swamp, shoal) identified through the vegetation or habitat analysis described above. Bird counts were made using binoculars (8 × 42) and telescopes (20-60 × 85) in the morning (0800–1000) or in the evening (1500–1730), when foraging activity normally peaks (Ringelman & Flake, 1980).

Although we did not ourselves undertook hydrological studies and we used previous work (Li *et al.*^2008^) to gain an understanding of surface and sub-surface hydrology, and its likely impact on the biological and human variables we measured. To assist with an understanding of human use of the Lake, and the delivery of ecosystem services, socio-economic, and cultural data were collected through analysis of peer-reviewed and “grey” literature (Shang *et al.*^2003^, Xing and Yang 1996). Socio-metric data were collected through interviews with differing groups of stakeholders, including the WNR administration, surrounding local communities, and people active in fisheries.

## Evaluation and discussion

### Vegetation & habitat

The land surrounding the WNR wetland consists mainly of lightly vegetated dunes, saline grass flats, farmland and recently planted woodland. Lake edges have extensive grassed areas; saline marshes develop where there is surface or near-surface water flow, and desert halophytes such as *Suaeda glauca, Tamarix ramosissima,Achnatherum splendens. Kalidium cuspidatum,Nitraria tangutorum,* are found on the drier edges of these marshes.

The plant communities of the wetland are simple in floristic and structure, being formed from a few highly dominant vascular plant species producing very high biomass yields. *T*he emergent reed swamp vegetation is dominated by 3-4 m high *Phragmites australis* (with 3-4m high *Typha latifolia,* and less frequentl*y T. minima* sometimes co-, or solely dominant).

Benthic vegetation is dominated by a mixture of *Potamogeton pectinatus, Myriophyllum spicatum* and an unidentified species of the macroalga *Chara*. In sites with better water quality other submerged macrophytes species occur (Table 1), but never in large numbers. There is evidence of significant phytoplankton and species of floating Chlorophyta, from which, given the shallow warm and highly eutrophic waters, algal blooms arise during the summer period. These blooms contribute to an increasing preponderance of de-oxygenation situations in the lake.

**Table 1:** **List of macrophytes inWuliangsuhai**

Emergent	Submerged
1 Gramineae Poaceae	3 Potamogetonaceae
(1) *Phragmites australis*	(4) *Potamogeton pectinatus*
2 Typhaceae	(5) *Potamogeton malaianus*
(2) *Typha latifolia*	(6) *Potamogeton zosterifolius*
(3) *Typha minima*	(7) *Potamogeton crispus*
	(8) *Potamogeton perfoliatus*
4 Najadaceae	
(9) *Najas* spp*.*	
	5 Haloragaceae
	(10) *Myriophyllum spicatum*
	6 Characeae
	(11) *Chara* spp*.*

### Bird population & migration pattern

**Figure 4:**
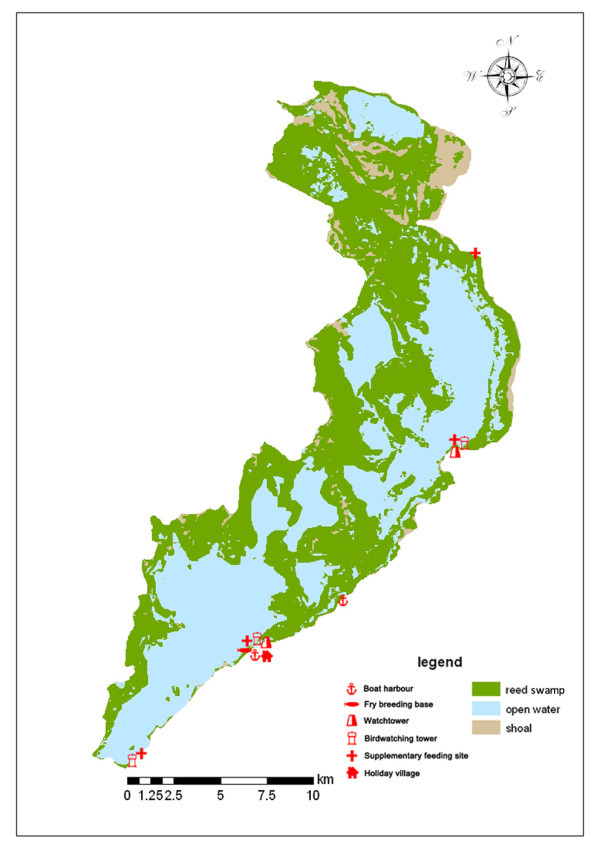
**Distribution of water bird population in summer (2009–2011).** DI is the distribution area with a density of 3000 water birds per km^2^; DII is a density of 2000 per km^2^; DIII is a density of 1000 per km^2^; “Breeding sites” refer to the breeding sites of *Cygnus.*

From the transects and plot surveys, we recorded 66 species, including 46 summer residents, 23 passing migrants and one vagrant. Comparing our data with census data held by the WNR administration, and previous studies (Xing and Yang 1996, Yang *et al.*^1999^, Pan *et al.*^2006^), we identified two sites with significant bird activity from spring – autumn (GSR and XHZ in Figure 4), and one (CSK in Figure 4) with significant bird activity in early spring, but little for the remainder of the year. More than 100,000 water birds roost in reed swamps and shoals (Figure 4), which are important for shelter in stormy conditions, while the open water is a good foraging area for ducks, geese and gulls. The migration exhibits a consistent pattern: birds start to arrive in March; March to April is the peak for immigration, followed by a breeding peak in late April to May, late August to October is the important period for emigration, and all birds have left by late October to early November. *Cygnus olor* starts breeding in mid-April and emigrates in late October.

Although not a major focus of this investigation, we noted vegetation communities surrounding the lake support viable populations of falcons, lapwings, turtledove and shrike.

In terms of habitat use by migratory birds, *Phragmites* swamps are good for foraging, nesting, moulting and sheltering, benthic vegetation is good for foraging, especially for ducks, swans and gulls, and the shoals are good for sheltering and roosting in storms or cold springs, for most species.

### Hydrology & water quality

Hydrology is the main abiotic determinant of the structure and composition of aquatic plant communities. (Brock and Casanova 1997, Bunn and Arthington 2002, Casanova and Brock 2000). The WNR is an important part of Ho-t'ao Irrigation and Drainage system, and it not only retains water for irrigation, but also plays important role in mitigating flood, and helps reduce saline intrusion from the groundwater. Of the lake’s water input, 96% is from the overflow and outwash from the irrigation system of the Ho-t'ao Plain (6.2xl0^8^ m^3^). The lake water outflows to the lake exits to the Yellow River. High water level is from September to October and the low water level from May to June. In most years the lake freezes in late October and thaws in late March, with the average frozen period of five months, and ice thickness in deeper water area reaches a maximum of 1.0 m (Duan *et a*l. 2005).

Current agricultural practices around the lake applied on average 550,000 tonnes of fertilizer and 1,500 tonnes of pesticides each year, and some of which eventually reach the lake. From 1970–2002, the total nitrogen load was 1088.59 tonnes per year, while the total phosphorous load was 65.75 tonnes per year (Shang *et al.*^2003^). The drainage and canals on the west shore of the lake, especially the 200 km main drainage canal, is the main source of nutrient input, bringing 85% of the water source for the WNR. In effect, the water quality of WNR depends on water quality arriving at and flowing through the main drainage canal, as well as the capacity of the reed swamp and macrophyte communities that reduce nitrogen and other pollutant loads.

### Human activity

Fisheries, tourism and reed harvesting are the three main industries historically and traditionally (Figure 4).

Local inhabitants harvest reeds from December to January during the period of maximum extent of lake ice, It is illegal under national regulations to undertake such activity in nature reserves but there does not appear to be any *a priori* reason why an allocation of reeds for harvest (possibly on a rotational system) should not be allowed. Reed harvesting is the most important industry, and financial resource, for the human population around WNR. The production increased from 9,770 tonnes in 1978 to 72,383 tonnes in 1989 and it continued to rise from 1993 after a slight decline from 1989 to 1993 (Duan *et al.*^2004^). Currently 130,000 tonnes per year are harvested, bringing an income of USD 6.16 million. In summer, macrophytes, particularly the abundant *Potamogeton pectinatus,* are also collected from submerged areas and used as animal fodder.

**Figure 5:**
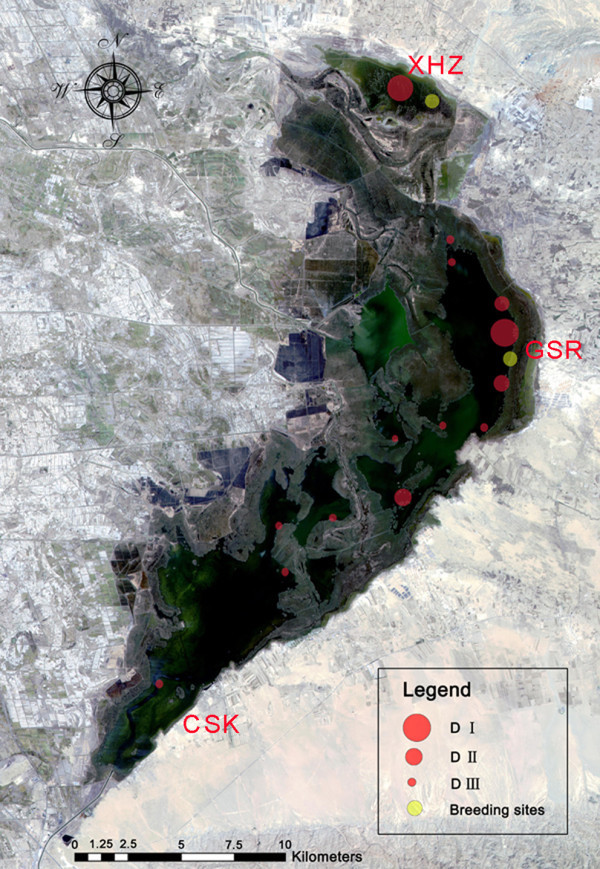
**Distribution of Wuliangsuhai wetland habitats & main human activities.** Green shows reed swamp, blue indicates open water, brown shows shoal.

Fishing is a traditional means of livelihood. Before 1958, the most abundant native fish in Wuliangsuhai Lake were *Cyprinus carpio, Leuciscus waleckii,Spualiobarbus curriculus, Silurus asotus,and Misgurnus anguillicaudatus*. The population of *Cyprinus carpio* especially suffered continual decline after 1960. In areas of the lake enjoying better water quality fry of four major Chinese carp (*Cyprinus carpio, Ctenopharyngodon idellus,Hypophthalmichthys molitrix and Aristichthysnobilis*) and Wuchang bream *Megalobrama amblycephala* were introduced from time to time to the lake from a fry breeding base (Figure 5) close to the boat harbour. However, shrinkage of the overall, and especially good quality, area of water, combined with over-fishing, resulted in a sharp decrease of fish resources, as shown by the decline in catch from 3575 tonnes in 1960 to 1000 tonnes in 2010.

WNR, and especially its lake, is a famous tourist destination known as the “Pearl beyond the Great Wall” and “Heaven of Birds”. As part of the tourist operations; there are packages including speedboat or water scooter activities, bird watching and fishing, most of which are undertaken close to the Boat Harbour (Figure 5). Around 100,000 tourists per year visit WNR, mostly from nearby Baotou city or Linhe city, representing an income of around USD 750,000. Most tourists come in summer and, of the tourist activities, speedboats and water scooters would seem likely to have the most negative influence on breeding birds, although this has not been adequately quantified.

After analysis of the relationship between key ecosystem services, its critical ecosystem components, and ecological processes, the comparison of effectiveness in maintaining ecosystem services by the two zoning models (the existing three-zone model, and the new zoning model) was made (Table 2).

**Table 2:** **Comparison between two zoningmodel based on analysisof CPS**

Key ecosystem SERVICES	Critical underlying ecosystem COMPONENTS	Critical underlying ecosystem PROCESSES	Three-zone model	New zonation
**SUPPORTING**				
S1. Providing habitats for 23 resident and 46 migratory birds	293 km^2^ shallow lake;	Hydrology: Freezing & melting	The habitat with most migratory birds (conservation target), were set as core zone	The habitat with most migratory birds (conservation target), were set as core zone.
	Habitat: Phragmites swamps	Primary production		
		Predation		
	Salt marshes	Breeding		Considering ecological processes, such as hydrological regime, predation and breeding, etc.
S2. Provides habitat for 11 aquatic fauna	293 km^2^ shallow lake; Water chemistry	Hydrological regime		
S3 Provides habitat for several fishes and benthos	Habitats: Open water with Macrophytes	Hydrological regime		
		Primary production of submerged plants		
	Water chemistry			
**REGULATING**				
S4. Water purification	Water chemistry –	Sedimentation, Connectivity	No special effort has been made	Risk-control zone was proposed to deal with external threats, such as over nutrient burden
	Nutrients,			
		Nutrient cycling, Carbon cycling, Oxidation reduction		
	Salinity & conductivity,			
	Sediment chemistry			
S5. Climate change mitigation	Plant communities	Primary production	No consideration	Dynamic zoning allows harvest of plants as industrial materials. Harvest itself improves primary production
		Sedimentation		
**PROVISIONING**				
S6. Fishery production of 1000 tonnes each year	Water chemistry –Salinity, Nutrients,	Irrigation & drainage currents	In experimental zone, some sustainable activities is allowed, but far from enough, especially in winter, the harvest season	Dynamic zoning allow for the harvest of reeds and macrophytes
	Biota: Macrophytes	Biological reproduction		
S7.130,000 tonnes of reeds for industrial use	Biota: Phragmites	Primary production		
S8. Animal fodder	Biota: Macrophytes	Primary production		
**CULTURAL**				
S9. Traditional harvesting and cultivation of fish and reed with historical and spiritual values	Fish: four major Chinese carp	Freezing & melting	All the harvest is forbidden in core and buffer zone according to Chinese Nature Conservation Law	Dynamic zoning allow tradtion reeds harvest, and fishing continues
	Biota: Phragmites			
S10. The site contributes to eco-tourism such as bird watching and boating	Birds:240 species	Bird migration	Limited eco-tourism allowed at the experimental zone	Through dynamic zoning, there is more space for tourism and scientific research in winter.
	293km^2^ shallow lake			
S11. The site is regionally important for scientific research and environment education	Birds: 240 species, mainly Migratory waterbirds and shorebirds.	Primary production		
		Biological migration		
		Pollutant transmit within the lake		

## Conclusion

### Proposed zoning

Using information derived from the CPS process, and given most of the surrounding land is semi-arid and has little conservation interest, we propose a refined zonation plan, differing from the existing by:

- reducing the surrounding farmland with little conservation value by 150 km^2^;
- slightly modifying the area of existing zones; and finally
- rename and suggestion of a new zone: risk-control zone

#### Core zone

**Figure 6:**
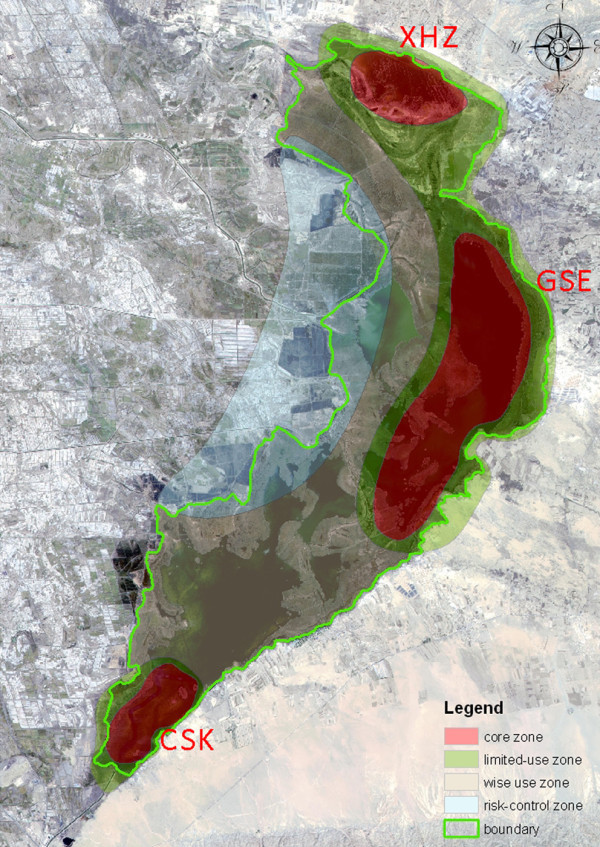
**Suggested spatial zonation of Wuliangsuhai Nature Reserve.**

The core zones are those of highest conservation value, with minimal human intervention so as to maintain ecosystem as integrity and minimise disturbance. In WNR, GSR, XHZ and CSK are suggested to be retained as core zones (Figure 6), since they are typical wetland landscapes with high numbers of visiting birds and low physical disturbance. The southern site (CSK, 12.77 km^2^), middle site (GSR, 55.31 km^2^), and northern site (XHZ, 15.87 km^2^), total (83.95 km^2^), take up 20.4% of the area (Figure 6). Key species such as *Cygnus olor, Platalealeu corodia* breed in these areas. *Ciconia nigra* and *Haliaeetus albicilla* also forage here.

#### Limited-use zone

Many authors (Dudley and Stolton 2008, Xu *et al.*^2007^) recently have drawn attention to the need for wetlands to be considered in a broader landscape, or wetscape (Bridgewater 2008), context, with appropriate links and connectivity developed between different landscape elements. To some extent, the proposed limited-use zone functions as a buffer in minimizing negative and external effects of human activities on the core areas, but also promotes connectivity between the zones and the wider landscape. Although the core zones should be fully and effectively (*i.e*. legally) protected, Limited-use zones also should have some protection, so that they can be designed to allow low-intensity sustainable use, helping maintain their function for both biological and cultural diversity conservation.

To achieve these objectives we suggest a 1km area around the core zones as a limited-use zone to assist with maintaining ecosystem integrity of ecological processes. This limited-use zone (Figure 6) also connects the core zones of GSR and XHZ, which are visited by local populations of swans, ducks, shore birds, and breeding grounds of *Cygnus olor*. The area of suggested limited-use zone is 92.51 km^2^, or 22.4% of the WNR.

#### Wise use zone

The Ramsar Convention defines wise use of wetlands as “the maintenance of their ecological character, achieved through the implementation of sound ecosystem approaches, within the context of sustainable development, to maintain environmental, economic and social sustainability in land use decisions, encourage compromises (“trade-offs“) between individual and collective interests.” (Ramsar Convention 2005). Using this definition we suggest the term wise use zone for areas with multiple land uses, but retaining a key role in delivering conservation through sustainable development.

Based on these concepts for zonation, our suggestion is to set the boat harbour, bird watching tower, fishery, and its adjacent water body as wise use zones (Figure 6), with an area of 132.47 km^2^, taking 32.1% of the total reserve area. These wise use zones are designed to maintain good conditions for fisheries, other harvesting traditions and wetland culture.

#### Risk-control zone

In semi-arid regions lake ecosystems are usually vulnerable, with a range of external threats being a major risk to maintenance of their ecological character. The main non-point pollution of Wuliangsuhai Lake is from irrigation water. Maintenance of water quality benefits vary with the size of the buffer, the flow pattern, vegetation type, percent slope, soil type, surrounding land use, pollutant types and dose, and precipitation patterns (Sheldon *et al.*^2005^). To relieve and remove ecological risk, we propose an area of drainage and canal systems in the west shore and its adjacent reed swamp should be set as a risk-control zone (Figure 6), with an area of 103.8km^2^, taking 25.1% of the area. Projects and programs such as pollution treatment, ecological restoration and biological conservation should be conducted in the zone to restore, and or maintain ecosystem function. The “experimental zone” in the existing zoning plan is largely sympatric with the wise use zone, although there is some overlap with the proposed risk-control zone.

The risk-control zone is of great importance. Agriculture activities outside WNR represent the main threat to the wetland ecosystem functioning, and thus service delivery. As a principal driver for wetland degradation, excessive fertilization contributing to the processes of eutrophication are directly or indirectly reinforced by climate change. And, for WNR, a major consequence of eutrophication is the extension of reed swamps, rapid growth and spreading of aquatic macrophytes, and the development of algal blooms, in particular, during warm summer periods. Remote sensing imagery has shown that from 1975 to 2001, the area within WNR dominated by reed swamp has increased by six-fold (Hou and Deng 2005). Shang *et al.* (2003) pointed out that the deposition of dead aquatic macrophytes into WNR lake was about 20.5 × 10^4^t DW per year. Historic data show that the area of open water in WNR has shrunk from 660 km^2^ in 1950s into 270 km^2^ in 2000 (Yu 2003),In the absence of active management within 30 years the current WNR would seem destined to become completely covered by reed swamp; and thus inimical to most of the bird species for which the area is designated a reserve. The risk-control zone is a new effort to deal with such kind of ecological risk.

**Figure 7:**
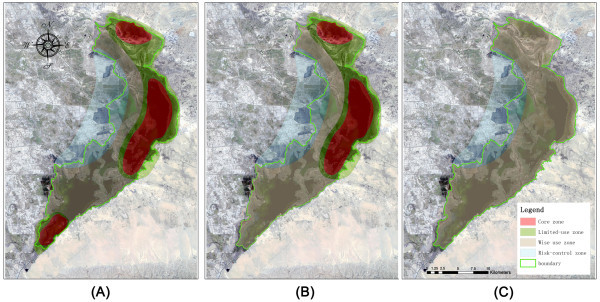
**Suggested Temporal Zonation of Wuliangsuhai Nature Reserve.** Red shows the core zone, light-green shows the limited-use zone, brown is the wise use zone, and light-purple shows the risk-control zone. The Wuliangsuhai wetland boundary is depicted by a solid green line. Figure 7**A** is the zonation proposed for March; Figure 7**B** is the zonation proposed for April to November; Figure 7**C** is the zonation proposed for December to February.

We have established that four spatial zones provide the maximum conservation benefit for the key objective of managing for migratory birds during their period of residency. However, we also advocate viewing those zones through a temporal lens, and modifying management efforts appropriately by varying the zonation structure and consequent management or monitoring efforts on an annual basis. We are able to prescribe this dynamic zoning structure for WNR on the basis of the adequacy and extent of available data, as following:

The four zones are especially effective from March to November (Figure 7A; Figure 7B) In March, however, the southern part starts melting and the early arrival migratory birds forage in the CSK core zone, which can be regarded thus as the key core zone for that period of the year (Figure 7A); while from April to October, most birds are resident in the XHZ and GSE core zones (Figure 7B). However, in ‘winter’ (December to following February), the whole lake is iced-over and all the birds emigrate; therefore a two zone structure - wise use and risk-control - are suggested for WNR during that period to allow for more efficient conservation effort (Figure 7C).

While the zones described above are presented as fixed spatial entities, the highly dynamic nature of WNR suggests a role for temporal as well as spatial zonation. Ecosystem management that allows for different management strategies to be expressed temporally helps achieve clearly enunciated conservation targets. As we have described the overall target for WNR – conservation and maintenance of migratory wetland bird populations - is seasonal in nature. Learning from nature and seizing the “right” time to maximise conservation is essential to developing an efficient management system. Accordingly, we suggest a system of dynamic zoning on an annual basis would further improve the effectiveness of WNR management. The premise of such dynamic zoning assumes a deep understanding of ecological processes at spatial and temporal scales, which we believe exists for WNR.

Despite many studies carried out to select or determine the shape, size and the optimal placement of nature reserves ( Blouin and Connor 1985, Buckley 1982, 1967, Higgs 1981, Higgs and Usher 1980, Li et al. 1999, Mac Arthur and Wilson 1967, Margules *et al*. 1982, Usher 1986), few practical studies for designing the interior structure of nature reserves, especially wetland reserves, have been published. Although no quantitative comparison has been made to the effectiveness of those different zoning patterns, this is the first endeavour to improve the existing zoning of WNR. It is quite true that each wetland has its own ecological characters. However, many of the wetlands are characteristic of dynamic change in vegetation, hydrology, migratory water bird population, *etc*. Therefore, the process to ecological character description and the zonation we propose for WNR would probably serve as an example, or new alternative to improve the basis for zonation establishment, which may have wide applications for wetland reserves that characterized by seasonal hydrological cycle, migration ground for birds, as well as under strong human use. Such zonation, taking account of human activity as well as that of wildlife, can help resolve the conflict between the objectives for nature conservation and community aspirations, and promote effective conservation and sustainable development for both nature and society.
